# Characterization of a Novel *Fibroblast Growth Factor 10* (*Fgf10*) Knock-In Mouse Line to Target Mesenchymal Progenitors during Embryonic Development

**DOI:** 10.1371/journal.pone.0038452

**Published:** 2012-06-13

**Authors:** Elie El Agha, Denise Al Alam, Gianni Carraro, BreAnne MacKenzie, Kerstin Goth, Stijn P. De Langhe, Robert Voswinckel, Mohammad K. Hajihosseini, Virender K. Rehan, Saverio Bellusci

**Affiliations:** 1 Member of the German Center for Lung Research, Excellence Cluster Cardio-Pulmonary System (ECCPS), Universities of Giessen and Marburg Lung Center (UGMLC), Giessen, Hessen, Germany; 2 Developmental Biology and Regenerative Medicine Program, Saban Research Institute of Childrens Hospital Los Angeles, University of Southern California, Los Angeles, California, United States of America; 3 Division of Cell Biology, Department of Pediatrics, National Jewish Health, Denver, Colorado, United States of America; 4 Department for Lung Development and Remodelling, Max-Planck-Institute for Heart and Lung Research, Bad Nauheim, Hessen, Germany. Member of the German Center for Lung Research; 5 School of Biological Sciences, University of East Anglia, Norwich, United Kingdom; 6 Department of Pediatrics, Harbor-UCLA Medical Center, Los Angeles Biomedical Research Institute at Harbor-UCLA, David Geffen School of Medicine at University of California Los Angeles (UCLA), Torrance, California, United States of America; Comprehensive Pneumology Center, Germany

## Abstract

Fibroblast growth factor 10 (Fgf10) is a key regulator of diverse organogenetic programs during mouse development, particularly branching morphogenesis. *Fgf10*-null mice suffer from lung and limb agenesis as well as cecal and colonic atresia and are thus not viable. To date, the *Mlcv1v-nLacZ-24* transgenic mouse strain (referred to as *Fgf10^LacZ^*), which carries a *LacZ* insertion 114 kb upstream of exon 1 of *Fgf10* gene, has been the only strain to allow transient lineage tracing of *Fgf10*-positive cells. Here, we describe a novel *Fgf10^Cre-ERT2^* knock-in line (*Fgf10^iCre^*) in which a Cre-ERT2-IRES-YFP cassette has been introduced in frame with the ATG of exon 1 of *Fgf10* gene. Our studies show that Cre-ERT2 insertion disrupts *Fgf10* function. However, administration of tamoxifen to *Fgf10^iCre^*; *Tomato^flox^* double transgenic embryos or adult mice results in specific labeling of *Fgf10*-positive cells, which can be lineage-traced temporally and spatially. Moreover, we show that the *Fgf10^iCre^* line can be used for conditional gene inactivation in an inducible fashion during early developmental stages. We also provide evidence that transcription factors located in the first intron of *Fgf10* gene are critical for maintaining *Fgf10* expression over time. Thus, the *Fgf10^iCre^* line should serve as a powerful tool to explore the functions of *Fgf10* in a controlled and stage-specific manner.

## Introduction

Fibroblast growth factor 10 (Fgf10) signals through its epithelial receptor Fgfr2b and is critical for branching morphogenesis in the mouse embryo. Loss of function of either *Fgf10* or *Fgfr2b* by genetic deletion leads to total failure of lung and limb formation as well as cecal and colonic atresia [1], [2], [3], [4], [5]. In addition to its vital role during development, *Fgf10* over-expression attenuates bleomycin-induced pulmonary fibrosis in mice [6].

Using enhancer-trap strategies, the *Mlcv1v-nLacZ-24* transgenic mouse line (hereby referred to as *Fgf10^LacZ^*) was characterized. The *LacZ* cassette was mapped and found to be integrated within regulatory elements lying 114 kb upstream of the endogenous *Fgf10* coding sequence [7]. *LacZ* expression pattern observed in these mice closely mimics *Fgf10* expression in the heart, lung, gut, mammary glands and brain [4], [5], [7], [8], [9], [10], [11]. The *Fgf10^LacZ^* mouse reports *Fgf10* expression and also results in a *Fgf10* hypomorph as demonstrated by the phenotype of *Fgf10^LacZ/−^* (one copy of *Fgf10* null and one copy of *Fgf10^LacZ^*) embryos. These embryos display decreased lung branching [12] as well as mammary gland [10] and limb abnormalities (Hajihosseini and Bellusci, unpublished results) consistent with decreased *Fgf10* expression.

**Figure 1:**
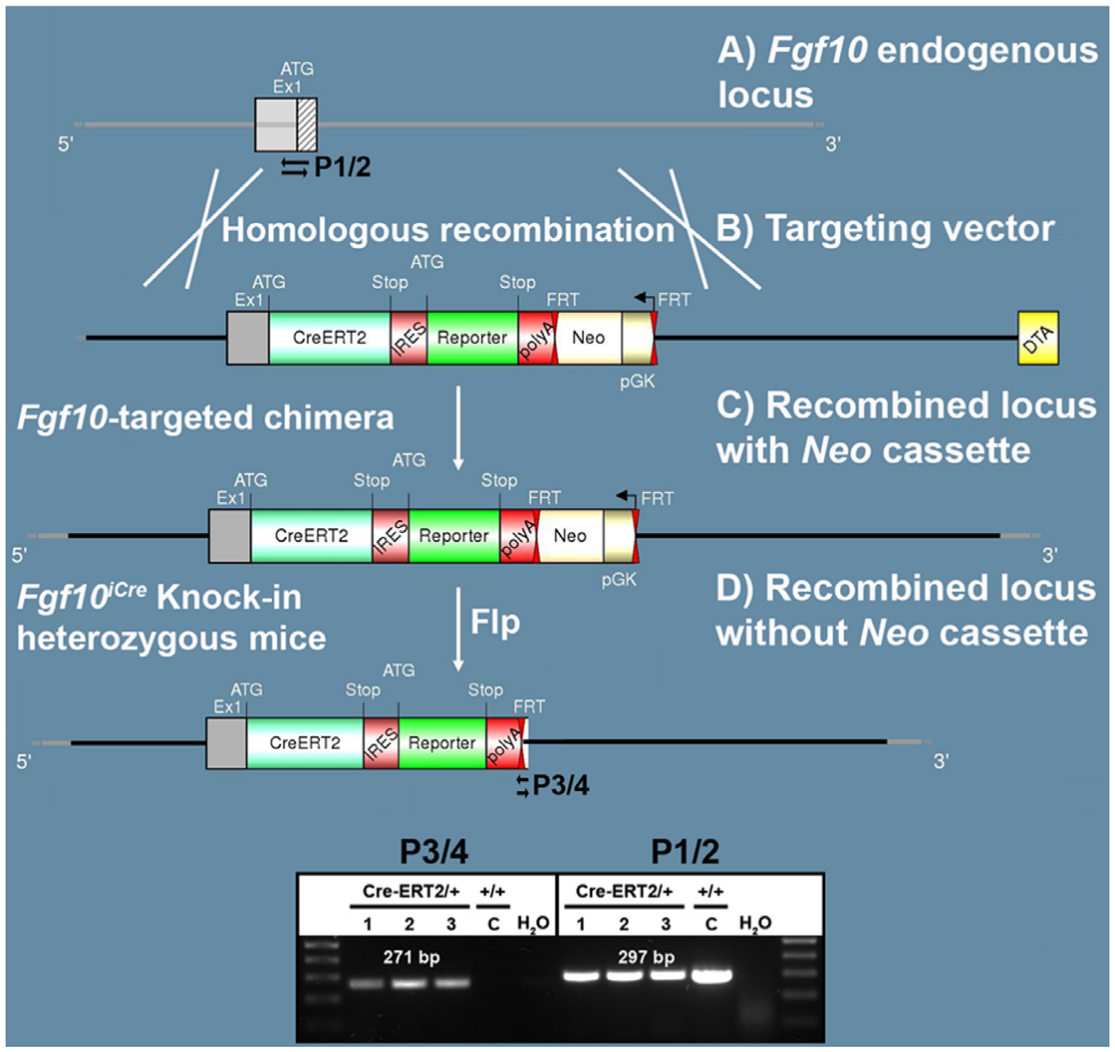
Generation of recombinant *Fgf10^iCre^* locus. (**A, B**) *Cre-ERT2-IRES-eYFP-Neo* construct was introduced in frame with ATG of exon 1 by homologous recombination. Genes coding for Neomycin resistance (*Neo*) and Diphtheria Toxin A were used for positive and negative selection respectively. (**C**) Chimeras carrying the *Neo* cassette were crossed with C57BL/6J mice ubiquitously expressing Flp recombinase to generate heterozygous knock-in mice lacking the *Neo* cassette (**D**). Primers used for genotyping are shown as black arrows. Note that primers P1 and P2 detect the wild-type locus (297 bp band) whereas primers P3 and P4 detect the recombined locus (271 bp band). C: control; DTA: Diphtheria Toxin A; pGK: Phosphoglycerate kinase promoter.

Until recently, the *Fgf10^LacZ^* transgenic line has been the only available tool that can be used to transiently trace *Fgf10*-positive cells in mice. Here, we describe a novel *Fgf10^iCre^* knock-in line where a tamoxifen-inducible Cre recombinase (Cre-ERT2-IRES-YFP) has been inserted in frame with the start codon of exon 1 of the endogenous *Fgf10* gene (Fig.1). Our validation studies show that *Fgf10^iCre^* is a loss of function allele for *Fgf10*. At the heterozygous state, this line is viable and fertile whereas the homozygous state is lethal. By crossing this line with a *Tomato^flox^* reporter line, we show that *Fgf10*-positive cells are specifically labeled upon tamoxifen administration in many tissues during embryonic and postnatal stages. Moreover, we demonstrate that crossing *Fgf10^iCre^* driver line with responder lines carrying ‘*floxed’* genes allows inducible conditional gene inactivation, but to a limited extent. This is because the expression of *Cre* from the recombined *Fgf10* locus reveals mosaicism in the labeled tissues and tends to decline with developmental progression. This is probably due to disruption of regulatory elements downstream of exon 1 where the cassette has been initially inserted. The mosaic recombination pattern observed in this genetic model is a valuable tool for clonal analysis and lineage tracing of *Fgf10*-positive cells.

## Materials and Methods

Targeting strategy of the endogenous *Fgf10* locus to generate *C57BL/6-Fgf10Cre-ERT2-YFP/J* knock-in mice.

5′ and 3′ homology regions (10.3 kb) for *Fgf10* were amplified by PCR using DNA from a *Fgf10*-specific BAC clone derived from a BAC library of 129Sv/AB2.2 mouse strain (Wellcome Trust Sanger Institute). The isolated sequences were used to design the targeting construct. The linearized targeting construct was transfected into TVB2 mouse ES cells (following the electroporation procedure: 5×10^6^ ES cells in presence of 40 µg of linearized plasmid, 260 Volt, 500 µF). Positive selection was started 48 hours after electroporation by addition of 200 µg/mL of G418. G418-resistant colonies were selected based on cell growth and morphology. One electroporation session was performed and a total of 209 clones were isolated and amplified in 96-well plates. Duplicates of 96-well-plates were made; one copy was frozen down and the other copy was amplified on gelatine to be used for genomic DNA preparation. Clones were screened by PCR for homologous recombination and verified by Southern blotting. Altogether, PCR, sequencing and Southern blot screening allowed the characterization of 10 recombined clones, 7 of which were suitable for blastocyst injection. Recipient blastocysts were isolated from pregnant C57BL/6J females (Health status VAF – Virus Antibody Free). Based on screening results and morphological criteria, ES cell clones were injected into C57BL/6J blastocysts. Injected blastocysts were then re-implanted into OF1 pseudo-pregnant females and allowed to develop to term. Four of the highly chimeric males generated were selected to breed with C57BL/6J Flp-deleter mice (health status SOPF – Specific and Opportunist Pathogen Free) to allow the germline excision of the neomycin selection cassette.

### Genotyping

Two primer pairs were used to genotype *Fgf10^iCre^* knock-in mice. Primers P1 (5’-AGCAGGTCTTACCCTTCCAGTATGTTCC-3’) and P2 (5’-CTCCTTGGAGGTGATTGTAGCTCCG-3’) were used to detect the wild-type allele (297 bp band) whereas primers P3 (5’-CAAACCCCAAAAGAACAGCTTTGTGTAC-3’) and P4 (5’-GACATTTGAGTTGCTTGCTTGGCACT-3’) were used to detect the knock-in allele (271 bp band). The PCR program consists of a denaturation step at 94°C for 2 min, followed by 35 cycles of denaturation (94°C for 30s), annealing (65°C for 30s) and extension steps (68°C for 300s). The program ends with a completion step at 68°C for 480s. Each PCR tube contains 2.6 U of Expand Long Template Polymerase in 5 µL of Reaction buffer 3 (Roche Applied Science, Mannheim, Germany), 15 pmol of each primer, 0.5 mM dNTPs and 10 ng of genomic DNA in a final volume of 50 µL.

### Mice and Tamoxifen Administration

*Tomato^flox/flox^* reporter mice (B6;129S6-*Gt(ROSA)26Sor^tm9(CAG-tdTomato)Hze^*/J) [13] were purchased from Jackson lab and *Fgf10^flox/flox^* mice [14], [15] were a kind gift from Professor Suzanne L. Mansour (University of Utah, USA). Embryonic day 0.5 (E0.5) was assigned to the day when a vaginal plug was detected. Mice were housed in an SPF environment. Animal experiments were approved by the Regierungspraesidium Giessen (Project ID763, approval number RP GI/20-Nr38/2011, 548_GP) and by the Animal Research Committee at Children’s Hospital Los Angeles (Protocol number 31-11) in strict accordance with the recommendations in the Guide for the Care and Use of Laboratory Animals of the National Institute of Health. The approval identification for Children’s Hospital Los Angeles is AAALAC A3276-01.

Tamoxifen stock solution was prepared by dissolving tamoxifen powder (T5648, Sigma, Schnelldorf, Germany) in corn oil at a concentration of 20 mg/mL at room temperature. Pregnant females carrying *Fgf10^iCre/+^; Tomato^flox/+^* embryos received a single intra-peritoneal (IP) injection of 0.075 mg tamoxifen per gram of body weight. For continuous tamoxifen exposure, pregnant females were fed tamoxifen-containing food (0.4 g of tamoxifen per kg of food) (Altromin, Lage, Germany). Dissected embryos were examined using Leica M205 FA fluorescent stereoscope (Leica, Wetzlar, Germany) and images were acquired using Leica DFC360 FX camera. Cecum lengths were measured using Leica’s LAS AF software and digit peripheries were measured using Image J software (NIH, USA). Figures were assembled in Adobe Photoshop CS5.

### X-Gal Staining

X-Gal staining was performed as previously described [16]. Briefly, *Fgf10^LacZ/+^* lungs were dissected out in Hank’s solution (HBSS) from E18.5 embryos, shortly fixed in 4% PFA, washed in PBS and incubated with LacZ buffer solution for 10 min. Then, they were incubated with LacZ buffer solution containing 40 mg/mL X-Gal (Sigma) at 37°C overnight.

### Quantitative Real-time PCR and Statistical Analysis

Freshly isolated embryos and lungs were lysed and RNA was purified using RNeasy kit (Qiagen, Hilden, Germany). E11.5 and E13.5 embryos were homogenized using QiaShredder columns (Qiagen) whereas lungs from older tissues were homogenized using Bullet Blender Blue (Next Advance, NY, USA). 1 µg of RNA was used for cDNA synthesis using Quantitect Reverse Transcription kit (Qiagen). Primers and probes for *Fgf10*, *Cre* and *β-actin* were designed using Universal ProbeLibrary Assay Design center (Roche Applied Science, available online at https://www.roche-applied-science.com/sis/rtpcr/upl/index.jsp?id=UP030000). More details about the used primers and probes can be found in Table S1. Quantitative real-time PCR (qPCR) was performed using LightCycler 480 real-time PCR machine (Roche Applied Science). Samples were run in triplicates using *β-actin* as a reference gene and the ΔΔCT method was used for relative quantification. Data were assembled using GraphPad Prism software (GraphPad Software, USA) and statistical analyses were performed using Student’s t-test (for comparing two groups) or One-way ANOVA (for comparing three or more groups). Data were considered significant if *P*<0.05.

## Results

### Generation of Fgf10^iCre^ (C57BL/6-Fgf10Cre-ERT2-YFP/J) Driver Line

129Sv ES cells were electroporated with a targeting vector containing the 5′ end of exon 1 of *Fgf10* open reading frame (Fig.1A, B). Immediately downstream of the start codon is the coding sequence of a tamoxifen-inducible form of Cre recombinase (Cre-ERT2) [17], followed by IRES sequence, enhanced YFP and Neomycin-resistance gene (*Neo*) respectively. Resistant ES cell clones were selected, screened by PCR and then verified by Southern blotting. Selected ES clones were injected into C57BL/6J blastocysts to generate chimeric pups (Fig. 1C). Chimeras were then crossed with C57BL/6J mice ubiquitously expressing Flp recombinase to generate heterozygous *Fgf10^iCre^* knock-in mice where the Neo cassette was totally excised (Fig. 1D).

### Insertion of the Cre-ERT2 Cassette in Exon1 Results in *Fgf10* Loss of Function

**Figure 2:**
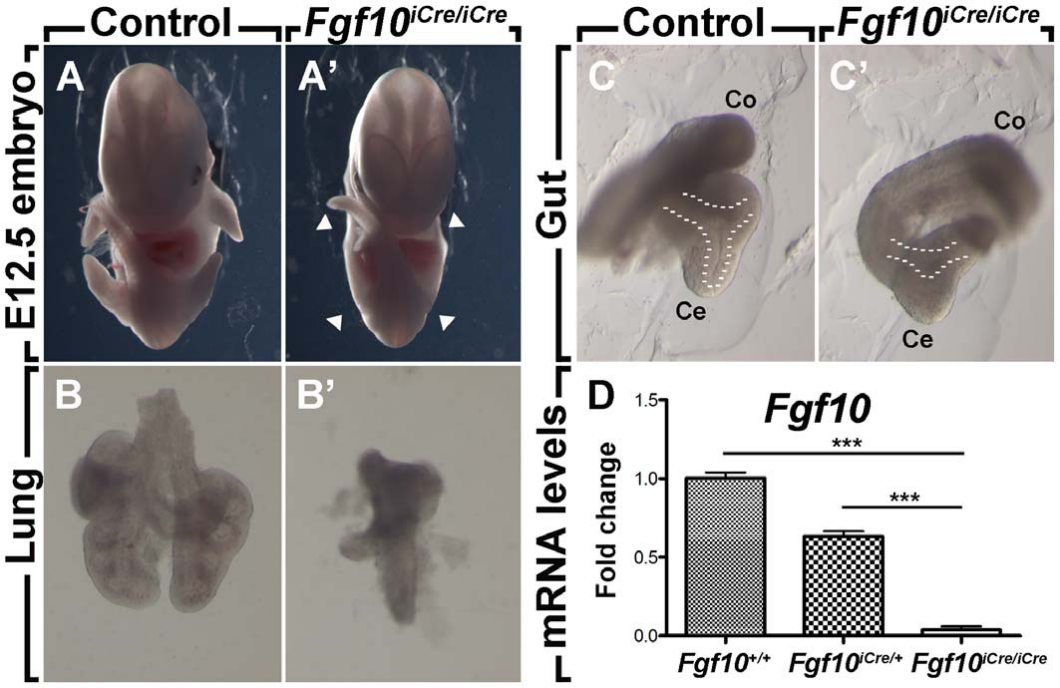
*Fgf10^iCre^* is a null allele for *Fgf10*. E12.5 *Fgf10^iCre/iCre^* embryos show agenesis of the limbs (A, A’) and lung (B, B’) as well as cecal and colonic atresia (C, C’). Arrowheads indicate sites of limb agenesis and dashed lines mark the epithelium in the cecum. *Fgf10^iCre/+^* embryos were used as controls. (**D**) *Fgf10* relative mRNA levels as quantified by qPCR. *Fgf10^iCre/iCre^* embryos (*n = 4*) express minimal *Fgf10* levels compared to *Fgf10^iCre/+^* (*n = 5*) and *Fgf10^+/+^* embryos (*n = 5*). Data are shown as average values ± SEM. * *P*<0.05; *** *P*<0.0001. Ce: cecum; Co: colon.

To determine whether the insertion of Cre-ERT2 in the endogenous *Fgf10* locus led to loss of function of *Fgf10*, *Fgf10^iCre^* heterozygous animals were crossed together and embryos were harvested at E12.5. *Fgf10^iCre^* homozygous embryos suffered from lung and limb agenesis in addition to cecal and colonic atresia, consistent with complete loss of function of *Fgf10* (Fig. 2A–C; *n = 4*; penetrance = 100%). *Fgf10^iCre/+^* embryos were used as controls. qPCR revealed minimal expression levels for *Fgf10* in *Fgf10^iCre/iCre^* embryos (Fig. 2D; *n = 4*) compared to *Fgf10^iCre/+^* (Fig. 2D; *n = 5*; *P*<0.0001) and *Fgf10^+/+^* embryos (Fig. 2D; *n = 5*; *P*<0.0001).

### Labeling of *Fgf10*-positive Cells

**Figure 3:**
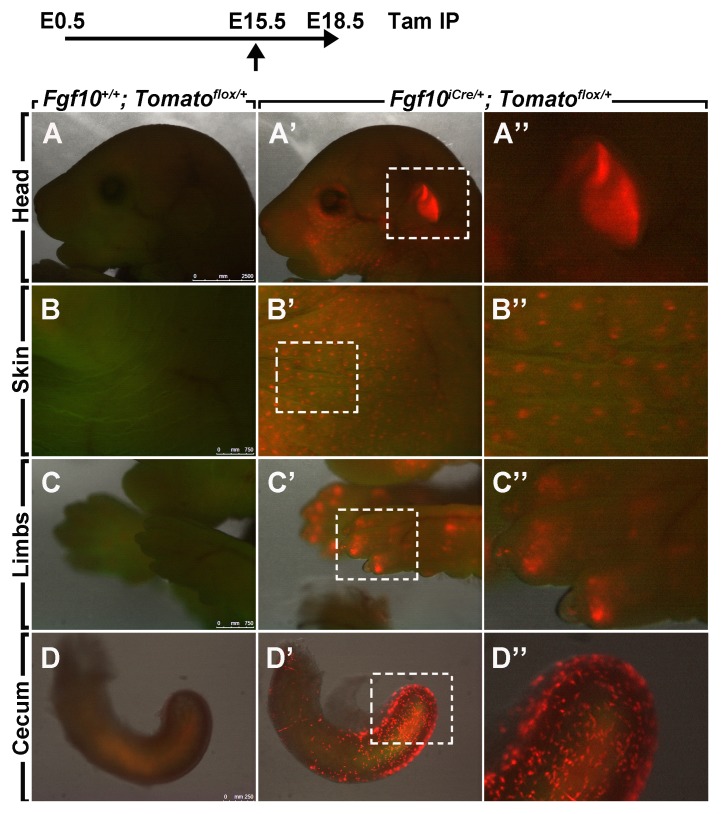
Tomato expression in E18.5 *Fgf10^iCre/+^; Tomato^flox/+^* embryos. Recombination was induced at E15.5 by a single IP injection of tamoxifen. Note the absence of Tomato expression in *Fgf10^+/+^; Tomato^flox/+^* embryos (**A–D**). Tomato-positive cells are detected in the ear, skin, limbs and cecum (**A’–D’**). (**A”–D”)** Higher magnifications of dotted boxes in **A’, B’, C’, D’**. *n = 3*. Tam: tamoxifen.

**Figure 4:**
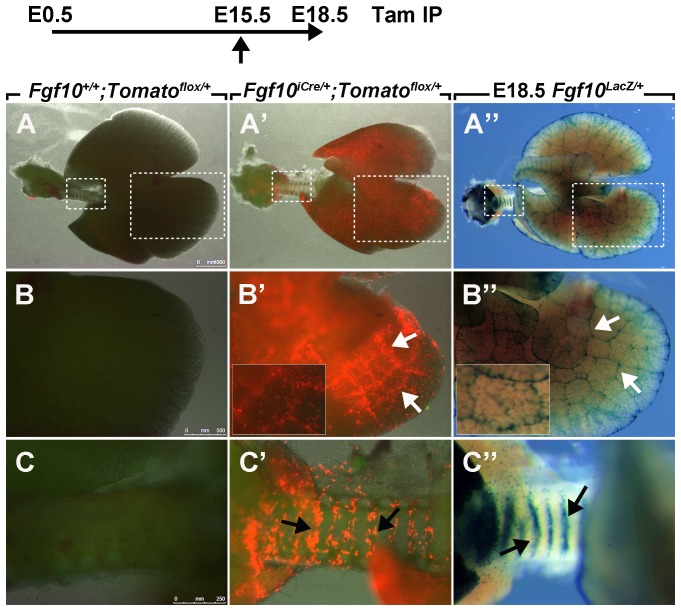
Tomato expression in E18.5 *Fgf10^iCre/+^; Tomato^flox/+^* lungs. Recombination was induced at E15.5 by a single IP injection of tamoxifen. Note the absence of Tomato expression in *Fgf10^+/+^; Tomato^flox/+^* lungs (**A;** *n = 3*). The regions in the dotted boxes are magnified in (**B**) and (**C**). Tomato-positive cells are observed in the lung mesenchyme and interlobular septae of *Fgf10^iCre/+^; Tomato^flox/+^* lungs (white arrows) (**A’, B’;** *n = 3*). Inset in B’ shows high magnification of interlobular septae. Labeled cells in the trachea arrange in ring-like structures (black arrows) (**C’;** *n = 3*). (**A”-C”;** *n = 8*) X-Gal staining of *Fgf10^LacZ/+^* lungs at E18.5. Inset in B” shows high magnification of interlobular septae. Tam: tamoxifen.

In order to test the recombinase activity of Cre-ERT2, *Fgf10^iCre^* heterozygous mice were crossed with *Tomato^flox/flox^* reporter mice. Pregnant females received a single IP injection of tamoxifen or corn oil at E15.5 and embryos were harvested at E18.5. No recombination was observed in *Fgf10^+/+^; Tomato^flox/+^* embryos (Fig. 3A–D; *n = 4*). Recombination was observed in the ears (Fig. 3A’, A”), skin (Fig. 3B’, B”), limbs (Fig. 3C’, C”), ceca (Fig. 3D’, D”) and lungs (Fig. 4A’-C’) of *Fgf10^iCre/+^; Tomato^flox/+^* embryos (*n = 3*; penetrance = 100%). Labeled cells in the skin were arranged in discrete spots (Fig. 3B’, B”). In the limbs, labeled cells were abundant at the tips of the digits as well as more proximal regions (Fig. 3C’, C”). In the cecum, however, elongated cells were labeled (Fig. 3D’, D”).

When examining the lungs from *Fgf10^iCre/+^; Tomato^flox/+^* embryos, Tomato-positive cells were detected throughout the mesenchyme (Fig. 4A’; *n = 3*). Interestingly, the signal was intense in interlobular septae (Fig. 4B’). In the trachea, Tomato-positive cells were arranged in ring-like structures (Fig. 4C’). X-Gal staining of *Fgf10^LacZ/+^* lungs at E18.5 revealed similar sites of *Fgf10* expression (Fig. 4A”-C”; *n = 8*). The expression of the YFP reporter from the IRES sequence could not be detected in any of the embryos. Tomato-positive cells were not detected in *Fgf10^iCre/+^; Tomato^flox/+^* lungs from corn oil-injected females (Data not shown; *n = 6*).

### Inducible Conditional Gene Inactivation

**Figure 5:**
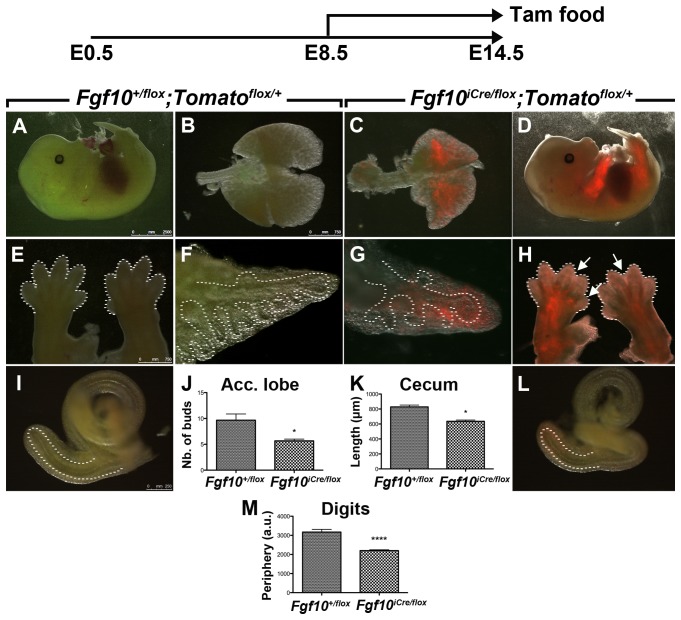
Inducible conditional *Fgf10* inactivation using *Fgf10^iCre^* driver line. Cre was activated by tamoxifen food from E8.5 to E14.5 in *Fgf10^iCre/flox^; Tomato^flox/+^* embryos. Labeled cells are present in the limbs, lung and cecum (**D, G, H, L**). Note the webbing of the digits in the forelimbs (arrows indicate webbing sites and dashed lines indicate peripheries) (**H vs. E, M**) and the hypomorph-like phenotype in the cecum (**L vs.** **I, K**). *Fgf10^iCre/flox^; Tomato^flox/+^* lungs show a deformed shape (**C** **vs.** **B**) as well as branching simplification (**G vs.** **F, J**). Dashed lines indicate the epithelium in the lung (**F, G**) and cecum (**I, L**). *n = 3*. Data are shown as average values ± SEM. * *P*<0.05; **** *P*<0.0001; Tam: tamoxifen.

To test the potential use of this line for gene inactivation studies, *Fgf10^iCre/+^; Tomato^flox/flox^* mice were crossed with mice carrying a *‘floxed’* version of *Fgf10* (*Fgf10^flox/flox^*) [14]. Cre activity was induced by tamoxifen food from E8.5 to E14.5. By using the *Tomato^flox^* reporter as readout of Cre activity, recombination was observed in the limbs, lungs and ceca (Fig. 5C, D, G, H, L; *n = 3*; penetrance = 100%). *Fgf10^iCre/flox^; Tomato^flox/+^* embryos suffered from diverse developmental abnormalities (*n = 3*; penetrance = 100%). A formation defect characterized by webbed digits was observed at the level of the forelimbs as compared to their *Fgf10^+/flox^; Tomato^flox/+^* control littermates. The phenotype was quantified by measuring the periphery of the digits (Fig. 5H vs. E, M; *n = 3*; *P*<0.0001). Hindlimbs did not show any obvious abnormalities. The lung showed an abnormal shape and suffered from branching simplification as illustrated by the reduced number of terminal buds in the accessory lobe (Fig. 5C vs. B, G vs. F, J; *n = 3*; *P*<0.05). The cecum, on the other hand, was shorter in *Fgf10^iCre/flox^; Tomato^flox/+^* embryos as compared to littermate controls (Fig. 5L vs. I, K; *n = 3*; *P*<0.05).

### Mismatch between *Cre* and *Fgf10* Expression Levels

**Figure 6:**
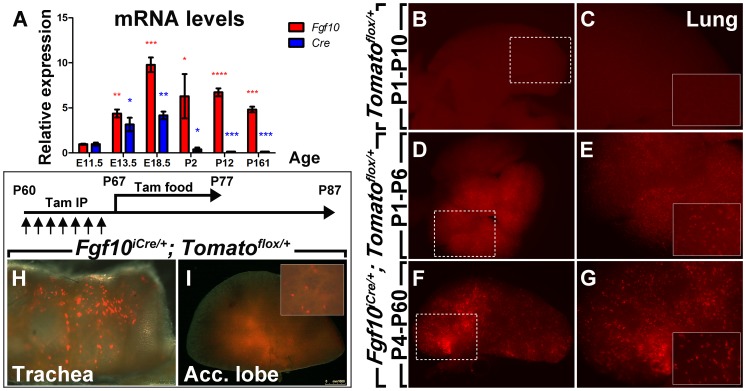
Mismatch between *Cre* and *Fgf10* expression levels in *Fgf10^iCre/+^* embryos. (**A**) *Fgf10* and *Cre* relative mRNA levels as determined by qPCR. For RNA preparation, whole embryos were used at E11.5 (*n = 5*) and E13.5 (*n = 3*) whereas lungs were used at E18.5 (*n = 7*), P2 (*n = 2*), P12 (*n = 4*) and P161 (*n = 3*). *Fgf10* expression levels increase throughout development and are maintained postnatally while *Cre* levels slowly increase throughout development and are minimal postnatally. (**B**) Induction in P1 *Tomato^flox/+^* pups reveals no recombination at P10 in the lung (*n = 2*). The area in the dotted box is magnified in (**C**). Inset in C shows high magnification with unlabeled cells. (**D-G;** *n = 4*) Induction in P1 and P4 *Fgf10^iCre/+^; Tomato^flox/+^* pups reveals recombination at P6 and P60 in the lung respectively. Insets in E and G show high magnification with labeled cells. (**H, I;** *n = 3*) Tamoxifen-induced recombination in lungs from *Fgf10^iCre/+^; Tomato^flox/+^* adult mice. Labeled cells are detected in the trachea and lung lobes. Inset in I shows high magnification with labeled cells. Data are shown as average values ± SEM. * *P<*0.05; ** *P<*0.01; *** *P<*0.001; **** *P<*0.0001.

In order to investigate the expression levels of *Cre* from the *Fgf10^iCre^* locus, timed-pregnant females carrying *Fgf10^iCre/+^* embryos were sacrificed at different developmental stages and *Fgf10* and *Cre* expression levels were assessed by qPCR by comparing mRNA abundance from different stages to E11.5. *Fgf10* expression levels progressively increased in *Fgf10^iCre/+^* embryos from E11.5 (*n = 5*) to E13.5 (*n = 3*; *P<0.01*) and E18.5 (*n = 7*; *P<0.001*) and were maintained postnatally at P2 (*n = 2*; *P<0.05*), P12 (*n = 4*; *P<0.0001*) and P161 (*n = 3*; *P<0.001*); however, *Cre* expression levels, from the same embryos, showed a slower increase from E11.5 (*n = 5*) to E13.5 (*n = 3*; *P<0.05*) and E18.5 (*n = 7*; *P<0.01*) and then dropped postnatally at P2 (*n = 2*; *P<0.05*), P12 (*n = 4*; *P<0.001*) and P161 (*n = 3*; *P<0.001*) (Fig. 6A). In spite of low *Cre* expression levels postnatally, single IP injections of tamoxifen at P1 or P4 led to significant recombination in *Fgf10^iCre/+^; Tomato^flox/+^* lungs at P6 and P60 respectively (Fig. 6D–G; *n = 4*; penetrance = 100%). Induction in P1 *Tomato^flox/+^* pups (single transgenics) did not reveal any recombination at P10 (Fig. 6B, C; n = 2). Last but not least, serial tamoxifen IP injections (one daily injection for one week), followed by a 10-day tamoxifen diet and 10-day normal diet, revealed recombination in the trachea and lung of *Fgf10^iCre/+^; Tomato^flox/+^* adult mice (Fig. 6H, I; *n = 3*; penetrance = 100%).

**Figure 7:**
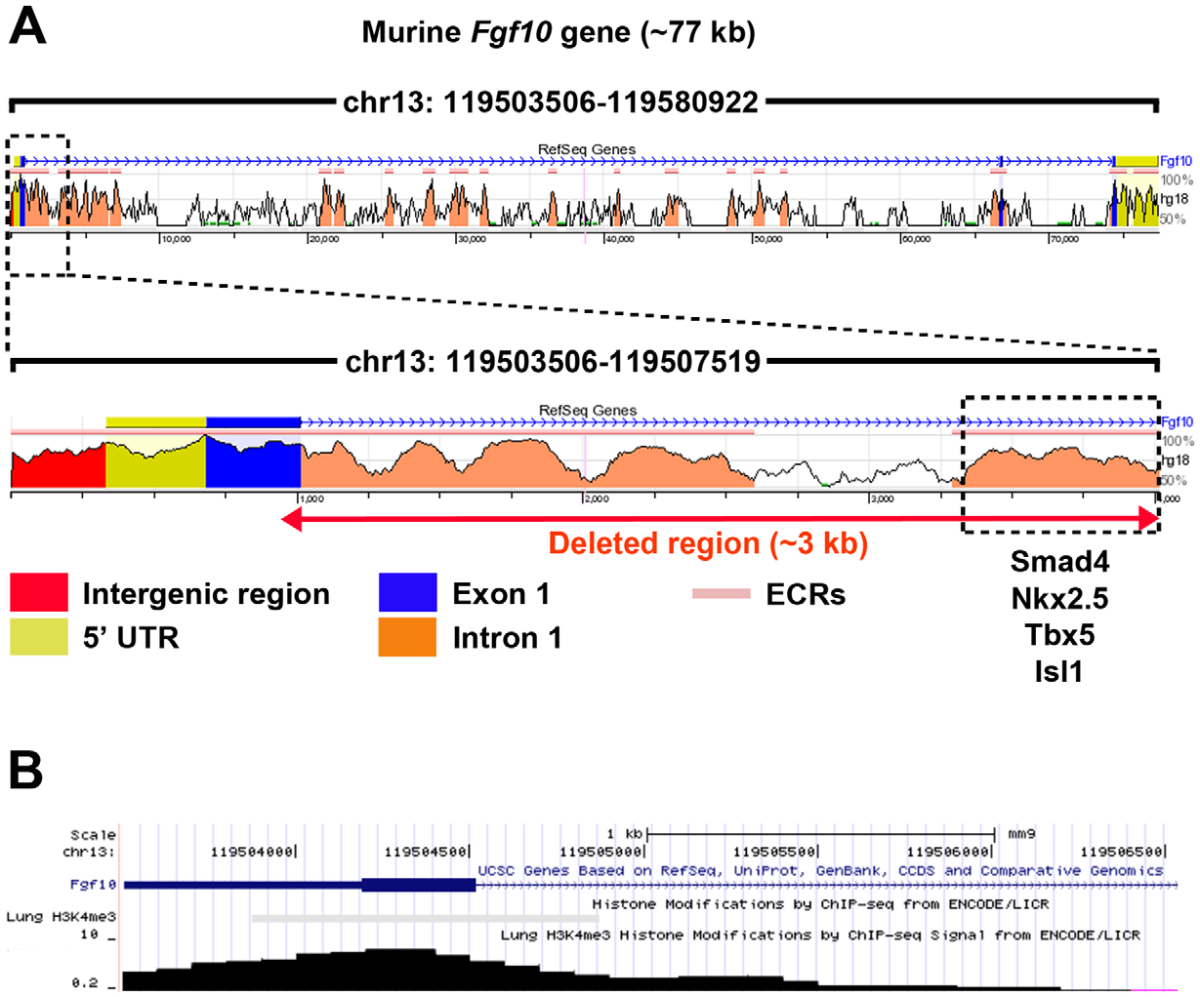
Bioinformatic screening for putative regulatory elements 3 kb downstream of *Fgf10* ATG. (**A**) The screening reveals regions that are rich in transcription factor binding sites conserved between mouse and human (as predicted by rVista; http://rvista.dcode.org). Lung-related transcription factor binding sites within the 3′ end of the deleted sequence are shown. (**B**) Screening for lung-related H3K4me3 modification sites (as predicted by UCSC Genome Browser; http://genome.ucsc.edu). A dense H3K4me3 modification site is predicted in the region overlapping exon 1-intron 1 boundary of *Fgf10* gene. UTR: Untranslated region; ECRs: Evolutionary conserved regions.

The 3 kb region downstream of *Fgf10* transcriptional start site, which was deleted upon homologous recombination, was analyzed for putative regulatory elements by comparing transcription factor binding sites conserved between mouse and human (using rVista, http://rvista.dcode.org). The analysis revealed the presence of several conserved transcription factor binding site-dense regions (Fig. 7A). Interestingly, the 716 bp stretch at the 3′ end of the deleted region contains putative binding sites for Smad4, Nkx2.5, Tbx5 and Isl1. Furthermore, the deleted region was analyzed for lung-related histone modifications using UCSC Genome Browser (http://genome.ucsc.edu). The analysis revealed the presence of a dense H3K4me3 (histone H3 trimethylated at Lys4) modification site overlapping exon 1-intron 1 boundary (Fig. 7B).

## Discussion

Fgf10 is a morphogen that is crucial for the embryonic development of many organs including the lung, duodenum, cecum, colon, pancreas, limb and mammary placodes. *Fgf10* knockout mice suffer from agenesis of the corresponding organs, thus limiting any real use of these mice in studying the behavior or lineage commitment of *Fgf10*-positive cells. On the other hand, due to the stability of β-Galactosidase, the *Fgf10^LacZ^* transgenic line allows non-inducible transient tracing of *Fgf10*-positive cells in many organs. However, the gold standard for an optimal lineage tracing tool relies on the inducibility of constitutive cell labeling via the controlled activation of Cre recombinase. Then, short- and long-term lineage commitment can be investigated. Therefore, the data obtained with the *Fgf10^LacZ^* line should always be validated using the proper Cre inducible line. Nevertheless, this line has helped identify the progeny of *Fgf10*-positive cells in the heart, lung, gut, mammary glands, brain [4], [5], [7], [8], [9], [10], [11] and limbs [Hajihosseini and Bellusci, unpublished results]. Thus, the *Fgf10^iCre^* knock-in line characterized in this paper represents a novel tool that bypasses the pitfalls associated with the *Fgf10^LacZ^* transgenic line. This is because *Fgf10*-positive cells are exclusively labeled upon tamoxifen injection, after which labeled cells can be traced and characterized. A previous report has shown that intra-peritoneally administered tamoxifen reaches effective levels in the embryonic circulation within 12 hours, after which it is cleared from the pregnant female within 12 hours [18]. This 24-hour time frame allows accurate labeling of *Fgf10*-positive cells.

In this study, we induced recombination in pregnant females carrying *Fgf10^iCre/+^; Tomato^flox/+^* embryos at E15.5. At E18.5, lineage-labeled cells were found in the ears of the embryos (Fig. 3A”) and this observation is consistent with previously published reports that describe the role of *Fgf10* in inner ear formation [19]. Moreover, labeled cells were observed in the skin (Fig. 3B”) where *Fgf10* is known to be expressed in the dermal papillae [20]. In the lung, labeled cells were dispersed throughout the mesenchyme (Fig. 4A’). However, the signal was more pronounced in interlobular septae (Fig. 4B’). Further studies need to be performed in order to explain this observation. As for the trachea, labeled cells were arranged in discrete ring-like structures (Fig. 4C’). This pattern is consistent with previously published reports about the role of Fgf10 in tracheal cartilage ring formation in mice [21], [22]. We also used *Fgf10^LacZ/+^* lungs from the same stage (E18.5) to validate our data. As expected, X-Gal staining of *Fgf10^LacZ/+^* lungs revealed similar sites of *Fgf10* expression (Fig. 4A”-C”).

For functional inactivation studies, we generated E14.5 *Fgf10^iCre/flox^; Tomato^flox/+^* embryos in which Cre had been activated since E8.5. By virtue of the embedded *Tomato^flox^* reporter, we were able to locate recombination sites and thus, we restricted our diagnostic to those specific sites. An abnormal phenotype, characterized by webbing of the digits, was observed in the forelimbs (Fig. 5H vs. E, M). This finding agrees with previous reports about the involvement of *Fgf10* misregulation in digit abnormalities such as syndactyly [23], [Al Alam and Bellusci, in revision]. Moreover, *Fgf10^iCre/flox^; Tomato^flox/+^* lungs showed clear developmental abnormalities characterized by a deformed shape as well as branching simplification (Fig. 5C vs. B, G vs. F, J). This phenotype is similar to that obtained by Abler et al. who used a general mesenchymal driver line *(Dermo1^Cre^)* to perform conditional gene inactivation of *Fgf10* [14]. Last but not least, our research group has previously demonstrated that the gut is a major site of *Fgf10* expression and that hypomorphic *Fgf10* embryos (*Fgf10^LacZ/−^*) suffered from cecal atresia [4]. Our data agrees with this finding as *Fgf10^iCre/flox^; Tomato^flox/+^* embryos suffered from a hypomorph-like phenotype at the level of the cecum upon partial loss of function of *Fgf10* (Fig. 5L vs. I, K).

One drawback of the *Fgf10^iCre^* line is the mismatch between *Cre* and *Fgf10* expression levels as examined at different developmental stages by qPCR (Fig. 6A). To understand the inefficient expression of *Cre* from the *Fgf10^iCre^* locus, we performed a bioinformatic analysis for the 3 kb deleted sequence downstream of ATG codon of exon 1 of *Fgf10* gene using online tools (Fig. 7). The analysis revealed the presence of H3K4me3 site overlapping exon 1-intron 1 boundary. This histone modification is known to be associated with the 5′ end of actively transcribed genes [24]. On the other hand, several conserved binding sites for lung-related transcription factors were detected, including binding sites for Smad4, Nkx2.5, Tbx5 and Isl1. Tbx5 is thought to directly control *Fgf10* expression in the lung mesenchyme [25]. Moreover, a recent report has shown that ISL1 regulates *FGF10* transcription within the second heart field by binding to an enhancer element in intron 1 of *FGF10* gene [26]. Altogether, these data suggest the presence of key regulatory elements in intron 1 critical for the maintenance of *Fgf10* expression over time. Nonetheless, this line allows to target a subset of *Fgf10*-positive cells after birth allowing to study the lineage commitment of these cells during homeostasis and during the repair process after injury.

Alternative strategies that would bypass potential consequences on *Cre* expression levels include fusion of Cre-ERT2 in frame with the ATG codon of *Fgf10* without deleting intronic sequences, thus preserving putative regulatory elements and matching *Cre* expression levels to endogenous *Fgf10*. Another approach would be the insertion of IRES-Cre-ERT2 in the 3' UTR downstream of *Fgf10* stop codon. In this case, *Fgf10* would still be expressed at physiological levels but *Cre* expression levels would depend greatly on the activity of the IRES element.

In conclusion, the *Fgf10^iCre^* knock-in line described in this paper is a novel tool that allows labeling and tracking of *Fgf10*-positive cells. The mosaic recombination pattern, as illustrated by the *Tomato^flox^* reporter, makes this line convenient for clonal analysis, especially when a partial loss of function approach is desired. This tool can potentially bring insight on the role of *Fgf10*-positive progenitor cells in development and repair after injury.

## Supporting Information

Table S1 **Primers and probes used for quantitative real-time PCR and designed using Roche’s Universal ProbeLibrary Assay Design center.**
(DOCX)
