# Supplementary material for: Characterization of a Novel Fibroblast Growth Factor 10 (Fgf10) Knock-In Mouse Line to Target Mesenchymal Progenitors during Embryonic Development
Source: PLoS One. 2012 Jun 13;7(6):e38452. doi: 10.1371/journal.pone.0038452 (PMC3374781; doi:10.1371/journal.pone.0038452)
Supplement: Table S1 — Primers and probes used for quantitative real-time PCR and designed using Roche’s Universal ProbeLibrary Assay Design center. (DOCX) [file pone.0038452.s001.docx]

## Supporting Information

**Table S1.**

| **Genes** |  | **Primers** | **UPL Probes** |
| --- | --- | --- | --- |
| ***Fgf10*** | **Forward** | 5’-ATGACTGTTGACATCAGACTCCTT-3’ | 63 |
|  | **Reverse** | 5’-CACTGTTCAGCCTTTTGAGGA-3’ |  |
| ***Cre*** | **Forward** | 5’-GTTTTGCCGGGTCAGAAAA-3’ | 87 |
|  | **Reverse** | 5’-GGCGCGAGTTGATAGCTG-3’ |  |
| ***β-actin*** | **Forward** | 5’-TGACAGGATGCAGAAGGAGA-3’ | 106 |
|  | **Reverse** | 5’-GCGTCAGGAGGAGCAATG-3’ |  |
